# Novel Inorganic Membranes Based on Magnetite-Containing Silica Porous Glasses for Ultrafiltration: Structure and Sorption Properties

**DOI:** 10.3390/membranes13030341

**Published:** 2023-03-15

**Authors:** Marina Konon, Elena Yu. Brazovskaya, Valery Kreisberg, Ekaterina Semenova, Irina G. Polyakova, Armenak Osipov, Tatiana Antropova

**Affiliations:** 1 Grebenshchikov Institute of Silicate Chemistry, Russian Academy of Sciences, 199034 St. Petersburg, Russia; 2 Chemistry Department, Lomonosov Moscow State University, 119991 Moscow, Russia; 3 Institute of Mineralogy, South Urals Federal Research Center of Mineralogy and Geoecology, Urals Branch of RAS, 456317 Miass, Russia

**Keywords:** porous glass, magnetite, sorption, methylene blue, inorganic membranes, phase-separated glass, leaching

## Abstract

Porous glasses (PGs) obtained from sodium borosilicate (NBS) phase-separated glasses via leaching are promising inorganic membranes. Introducing Fe_2_O_3_ into NBS glasses imparts ferrimagnetic properties due to magnetite crystallization. Leaching of such glasses leads to the formation of magnetic PGs with interesting electro-surface characteristics. This work aimed to investigate the process of obtaining magnetite-containing PGs from NBS glasses depending on silica content, using XRPD and Raman spectroscopy, studying the PG membranes’ structural characteristics and their sorption properties with respect to methylene blue (MB). Obtained PGs were characterized by a polymodal distribution of mesopores and a small number of micropores with specific surface area values of 32–135 m^2^/g and an average mesopore diameter of 5–41 nm. The kinetic data were analyzed using pseudo-first-order, pseudo-second-order, and intra-particle diffusion equations. The equilibrium isotherms were fitted with Langmuir, Freundlich, Temkin, and Dubinin-Radushkevich models. MB adsorption was found to be a complex process. The glass with the highest specific surface area demonstrated the maximum sorption capacity (10.5 mg/g). The pore size of PGs allowed them to be considered potential novel magnetic membranes for ultrafiltration.

## 1. Introduction

Membrane technology is an environmentally friendly and highly effective resource- and energy-saving technology. The use of membranes makes it possible to create economically efficient and low-waste techniques for processing the aqueous and non-aqueous solutions of inorganic and organic compounds, gas separation, wastewater treatment, etc. [1] One of the ways to classify the separation processes is by the size of the components needing to be separated. One of those processes is ultrafiltration, which is a well-established process for protein concentration, buffer exchange, and purification [2]. It is often used for the separation process in water applications [3,4] because ultrafiltration membranes are capable of removing macromolecules and colloidal solutes, microorganisms, and particulate matter more effectively than other conventional separation processes due to their pore size of 10–1000 nm [5,6].

The material the membranes are made of can be inorganic (metal, ceramic, glass, etc.) or organic (polymeric: films, tubes, hollow fibers). Polymeric membranes have been widely used in various industrial processes for many years, mainly due to their relatively low manufacturing costs and ease of molding and scaling. Such membranes, however, are not without quite serious drawbacks, such as limited strength, inability to withstand harsh chemical, thermal, or mechanical conditions, increased operating costs for cleaning or replacing the membrane after the fouling, and the need to dispose of them after use, which exacerbates environmental problems [1].

From this point of view, inorganic membranes, which are much more durable and have a lower environmental impact, will play an increasingly important role in various membrane separation processes, including the separation of gas mixtures, in the coming decades [7,8,9]. The most important advantages of inorganic membranes are their thermal, mechanical, biological, and chemical resistance, allowing them to be used to separate aggressive media and easily regenerated and disinfected by calcination or treatment with special solutions [1]. Inorganic membranes are also advantageous in brackish water treatment, seawater pretreatment, and high-temperature desalination, where high rejection above 99% is not a critical requirement. These types of industrial wastewater are normally discharged in large quantities and pose a great threat to the conventional wastewater treatment system due to their complex nature and chemical composition [9].

Due to the ability of inorganic membranes to be used under specific technological conditions (high temperature and pressure), they are not considered a replacement for polymeric membranes in existing applications but rather as materials that will find and satisfy new market opportunities for membrane separation processes [1,10]. Inorganic membrane penetration into the market will accelerate as their general performance improves; therefore, research in this area is an urgent and needed task from the point of view of ecology and energy saving [9,10].

Among inorganic membranes, the largest share comprises ceramic membranes, which are successfully used in various industries, but their employment is associated with certain limitations. In particular, because it is impossible to obtain pores with a sufficiently small diameter and narrow size distribution in them, one has to resort to an additional modification stage [1,11]. In this regard, membranes based on porous glass (PG) are promising inorganic membranes. PG is obtained by chemical leaching of phase-separated sodium borosilicate (NBS) glass with an interconnected structure formed during the phase separation process with a special heat treatment [12,13,14,15]. PGs are widely used as adsorbents (for example, in chromatography as substrates for adhesive or chemically bound stationary phases), separation membranes (for seawater desalination, emulsion separation, hemofiltration, and diafiltration), antiviral filters, catalyst carriers, matrices for composite materials, etc. [12,13,14,15,16]. PGs have a number of advantages: homogeneity of chemical composition, low level of foreign impurities, large specific surface of silica sorbent, thermal, chemical, and microbiological stability, and mechanical strength (During the long-term operation of PG membranes, their performance does not change [17,18]). PGs are the only type of porous adsorbents that, based on their structural features, make it possible to realize pore sizes in the entire IUPAC classification range [13]. Among various types of membrane materials, due to the possibility of varying the internal texture of pores, surface polarity, the ability to modify the surface due to the presence of silanol groups, and also due to the possibility of preparing stable porous glass bodies with variable shape and size (such as tubes, plates, and hollow fibers), PGs can be considered as an easily adaptable material for application in various fields of membrane technology [19,20,21,22,23,24,25].

For the longest time, the best-known industrial producer of PGs was Corning Inc. (Corning, NY, USA) with its famous glass product Vycor^®^ 7930 [26,27], which readily lent itself to research for various applications. Recently, however, Corning discontinued Vycor^®^ 7930 Glass production, and now a reliable alternative is in demand [28]. One of the alternatives could be SCHOTT CoralPor^®^ Porous Glass, a registered trademark of SCHOTT North America, Inc. (Duryea, PA, USA) [29]. Nevertheless, the research in the field of porous glasses has not stopped, and VYCOR^®^ 7930 is still used as a porous model body for emerging applications, such as separators in all-vanadium redox flow batteries and lithium-ion batteries [30,31], along with the new “home-made” compositions of PGs [32,33] for various modern materials.

Modification of PGs by impregnating them with different compounds is a well-established route of imparting PGs with new functional properties, such as photocatalytic [34], luminescent [35], magnetic [25], etc. Magnetic properties are especially interesting for membrane technology. Compared to their nonmagnetic counterparts, magnetic membranes have improved permeability, a higher degree of purification, and greater antifouling ability due to the vibration of magnetic nanoparticles that occurs when a magnetic field is applied [36,37,38,39,40]. However, impregnating PGs with iron oxides [25] is not the only way to obtain an inorganic membrane with magnetic properties. The introduction of Fe_2_O_3_ into the NBS glass batch containing 70 mol% SiO_2_ before melting was established to cause magnetite crystallization, which has ferrimagnetic properties [41,42,43,44,45]. Magnetite-containing PGs were used as a host matrix for multiferroic composite materials obtained by impregnating the pore space with ferroelectric phases [46,47,48]. It was demonstrated that adding Fe_2_O_3_ to the NBS glass composition also changes the electro-surface characteristics of resulting PGs. In particular, a positive region of the values of the electro-kinetic potential appears at pH < 4, which opens up new prospects for the application of high-silica PGs in various modern membrane and sorption technologies [49,50].

Consequently, the objective of this investigation was to study the possibility of obtaining porous membranes containing magnetite from NBS glasses with a lower silica content (55–65 mol% SiO_2_), as well as their sorption properties, using methylene blue (MB), which often serves as a model compound for the adsorption studies of organic contaminants in aqueous solutions due to its stability to heat, oxidizing agents, and biodegradation [51]. To our knowledge, MB adsorption studies on this type of membrane have not been performed yet.

## 2. Materials and Methods

### 2.1. Glass Synthesis

Glass with compositions 6Na_2_O-*x*B_2_O_3_-(86-*x*)SiO_2_-8Fe_2_O_3_, where *x* varies from 21 to 31 mol%, was synthesized using conventional melting according to the procedure described in detail in [52,53]. The glass batch was mixed from the reagent grade H_3_BO_3_ (Vekton, Saint Petersburg, Russia) and Na_2_CO_3_ (ECROS, Saint Petersburg, Russia), analytic grade Fe_2_O_3_ (LenReactiv, Saint-Petersburg, Russia), and SiO_2_ in the form of ground high-purity quartz glass (KV-glass, Russian state standard 15130-86, metal impurities ≤ 1 × 10^−2^ wt%, OH groups—(1.5–6) × 10^–2^ wt%). The glasses were melted in platinum crucibles in an electric furnace with SiC heating elements with constant stirring of the melt with a platinum stirrer at 1320–1500 °C in air for 2–3 h, depending on the concentration of SiO_2_. The glass melt was poured onto a heated steel plate and transferred to an electric muffle furnace for annealing (temperature 510–550 °C, duration—5 min). The leaching kinetics, gas adsorption, and sorption of MB study results for these iron-containing glasses were compared with a sodium borosilicate glass with a composition (mol%) of 6Na_2_O‧34B_2_O_3_‧60SiO_2_, synthesized in a similar way as described in [54].

After annealing, all glasses were additionally heat treated in a muffle furnace at 550 °C for 24 h to promote phase separation.

### 2.2. Chemical Analysis of Glasses

The compositions of the phase-separated glasses and PGs were determined using the following analytical chemistry techniques. Sodium and iron content was analyzed using standard techniques (accuracy: Na_2_O—±2 rel%, total iron content—±1 rel%, FeO—±4 rel%) [55,56,57]. The amount of B_2_O_3_ was estimated by potentiometry (accuracy ± 0.4 rel%) [58]. The SiO_2_ content was determined using the gravimetric method using the quinoline-silicon-molybdenum complex (accuracy ± 0.08 rel%) [59].

### 2.3. X-ray Powder Diffractometry (XRPD)

The diffraction patterns for phase-separated glasses and PGs were obtained in the DRON-3 unit (Scientific Production Association “Burevestnik,” Saint-Petersburg, Russia), CuK_α_ radiation. Crystalline compounds were identified by powder diffraction files using the PDF-2 database.

### 2.4. Raman Spectroscopy

Raman spectra for phase-separated glasses were recorded on samples in the form of plane-parallel polished plates on an iHR320 Horiba Jobin-Yvon spectrometer (Montpellier, France) equipped with an Olympus B × 41 optical microscope. The spectra were excited using a helium-neon laser (radiation wavelength 632.8 nm) with an optical output power of 15 mW.

### 2.5. Leaching Kinetics

The chemical durability and leaching kinetics were studied by leaching polished plates of phase-separated glasses with the size of 10 × 10 × 1 mm^3^ in an aqueous 3M HCl solution at boiling according to the procedure described in great detail in [42]. To evaluate the extraction leaching rate and the kinetics of the components (Na_2_O, B_2_O_3_, SiO_2_, Fe_2_O_3_) from the chemically unstable boron-rich phase of the glass during acid treatment (for 5 h), aliquots (15 mL) were taken 15, 30 min, 1, 2, 3, 4, and 5 h after placing the glass samples in boiling acid, to measure the concentrations of the components in the leaching solution. After leaching, the glass samples were washed in distilled water for 5 days at room temperature and then dried in a drying cabinet for 1 h at 120 °C.

The leaching rate was estimated by the experimental value of the number of components (*Q*_exp_, g) passing from the glass surface unit (*S*_0_, cm^2^) into the solution in a definite time. (*Q*_exp_/*S*_0_, g/cm^2^) values were compared with the theoretically possible ones (*Q*_calc_/*S*_0_, g/cm^2^). *Q*_calc_ for each component was calculated by multiplying the volumetric concentration of the component (*C*_v_, g/cm^3^) by the sample volume (*V*_0_, cm^3^). *C*_v_ was calculated using the glass composition (in wt%) and the density value (ρ, g/cm^3^) (Table 1). The density was determined by hydrostatic weighing in water at 20 °C (±0.005 g/cm^3^). The glass compositions in mol% and density values for the iron-containing glasses were previously published in [53].

Determination of the glass components concentration in the leaching solution was carried out by the analytical chemistry methods. The content of boron was determined using potentiometry (accuracy ± 0.4 rel%) [58], sodium content—using Flame Atomic Absorption Spectrometry (accuracy ± 2 rel%) [55], and the silicon and iron concentration was evaluated by spectrophotometric measurement (accuracy ± 10 rel%) [60].

The effective diffusion coefficients of Na, B, and Fe were calculated according to Equation (1), as described in detail in [42]:

D* = π*Q*^2^/(4*C*_1_^2^*t*), (1)

where D* is the effective diffusion coefficient (cm^2^/s); *Q* = *Q*_exp_/*S*_0_ (gram atom/cm^2^); *C*_1_—volumetric concentration of the component in glass (gram-atom/cm^3^) calculated from the composition (wt%) and the glass density (g/cm^3^); *t* is the time of leaching (s). The error in determining the D* values did not exceed ±0.7 × 10^−7^ cm^2^/s. As noted previously [42], due to the absence of a metastable immiscibility diagram for the Na_2_O-B_2_O_3_-SiO_2_-Fe_2_O_3_ system, and consequently, the lack of knowledge of the position of immiscibility isotherms and tie-lines, it is currently impossible to determine the compositions and volumes of coexisting phases, the density, *C*_1_, and the surface area of the chemically unstable boron-rich phase occupied on the sample surface. Therefore, the C_1_, composition, and density values for the whole glass were used in calculations. Hence, the D* values determined with the aforementioned assumptions can be considered as some reduced values.

### 2.6. Classical Gas Adsorption Method

The equilibrium nitrogen adsorption and desorption isotherms at liquid nitrogen temperature (77 K) in the PGs were obtained using Quantachrome NOVA 1200e (Quantachrome Instruments, Boynton Beach, FL, USA). Isotherms were measured within the relative pressure range p/p^0^ = 0.005–0.988, which made it possible to register mesopores (2–50 nm in size according to the IUPAC nomenclature [61]) and large micropores (1–2 nm). The analysis of nitrogen adsorption and desorption isotherms at 77 K was performed according to the modified Barret-Joyner-Halenda (BJH) method. Mesopores were recorded in the region of medium and high relative pressures in accordance with the mechanism of capillary condensation, and micropores were diagnosed in accordance with the mechanism of volume filling in the region of low relative pressures, using the dependence of partial pressure on the diameter of micropores, embedded in the software of the Quantachrome Instruments device, and found by the density functional theory (DFT method) [62,63]. The experimental equilibrium nitrogen adsorption and desorption isotherms at 77 K, which include both the region of medium and high relative pressures and the region of low pressures, were approximated by a five-modal equation with Boltzmann functions [62,63,64,65].

A gravimetric method also determined the pore volume (*V*_p_, cm3/g) and porosity (*W*, %). The measurement error for *W* was ±3%, for *V*_p_ ± 0.03 cm^3^/g.

### 2.7. Methylene Blue Adsorption Studies

Batch Adsorption Experiments. The adsorption properties of the bulk samples concerning MB were studied under static conditions from aqueous MB solutions with a concentration of 12 to 20 mg/L. The experiments were conducted in darkness at room temperature (25 ± 1 °C). Before sorption, all samples were dried at 120 °C for 24 h. To a weighed portion of the sample (20 mg), 20 mL of MB solution was added and stirred on a magnetic stirrer for the time necessary to plot the kinetic curves (from 1–24 h). After completion of the experiment, the solution was centrifuged. MB concentration was determined using UV-visible absorption spectroscopy (Shimadzu UV-2600/2700, Shimadzu Europa GmbH) by optical density at a wavelength of 246 nm. Each point in the kinetic curve was taken as the average value of the three measurements. Sorbent capacity, mg/g (amount of adsorbed substance), was determined by Equation (2):

(2) $$q_{t}=\frac{(C_{0}-C_{t})\;V}{m}$$

where *C*_0_ and *C_t_* are the initial concentration of the MB solution and at time *t* (mg/L); *V* is the volume of the MB solution (L); *m*—sample weight (g).

The study of the equilibrium adsorption of MB was carried out at the initial concentrations in the range of 8–20 mg/L. To do this, 20 mg of a sample was dispersed in 20 mL of an aqueous solution of MB of a given concentration. The experiments were carried out in a static mode in closed glass bottles with a volume of 50 mL with stirring for the time necessary to achieve adsorption equilibrium (up to 24 h). The samples were filtered, and the concentration of MB in the filtrate was determined as the arithmetic mean of three measurements.

Batch kinetic models. Three models were used to analyze the adsorption mechanism: the pseudo-first-order (PFO) Equation (3), the pseudo-second-order (PSO) Equation (4), and the intra-particle diffusion Equation (5). The kinetic expression for PFO, based on the capacitance of the solid, is expressed by the following Equation (3):

(3) $$q_{t}=q_{e}\times \left(1-e^{-kt}\right)$$

where *q_t_* is the sorption capacity at a time *t*, *q_e_* is the sorption capacity in equilibrium (mg/g), and *k*_1_ is the PFO reaction rate constant, min^–1^.

The PSO model is represented as follows:

(4) $$q_{t}=\frac{q_{e}^{\;2}\times k_{2}t}{1+k_{2}\times q_{e}}t$$

where *q_t_* is the sorption capacity at any time *t*, and *q_e_* is the sorption capacity in the equilibrium state (mg/g), *k*_2_ is the PSO rate constant (g/(mg min)).

The intra-particle diffusion model is usually used to determine the rate-controlling step in the porous structure, and its Equation is listed as follows:

(5) $$q_{t}=k_{id}\times t^{0.5}+C_{i}$$

where *C_i_* is (mg/g), a constant related to the thickness of the boundary layer (high values of *C_i_* indicate a high effect of the boundary layer), *k_id_* is the intra-particle diffusion rate constant (mg/g·min^0.5^), and *t*^0.5^ is the square root of the time [66,67].

Adsorption isotherm models. In the present study, the adsorption equilibrium data were analyzed by the Langmuir Equation (6), Freundlich Equation (8), Temkin Equation (9), and Dubinin-Radushkevich Equation (10) isotherm models. The parameters of the adsorption equations were calculated using the non-linear regression method using the OriginPro 8 program.

The Langmuir model can be expressed by Equation (6):

(6) $$q_{e}=\frac{K_{L}q_{m}C_{e}}{1+K_{L}C_{e}}$$

where *q_m_* is the maximum sorption capacity of complete monolayer coverage (mg/g), *C_e_* is the equilibrium concentration (mg/L), and *K_L_* is the Langmuir constant related to the free energy of adsorption (L/g) [66].

The efficiency of adsorption can be estimated from a dimensionless constant called the separation factor *R_L_* according to Equation (7):

(7) $$R_{L}=1/\left(1+K_{L}\times C_{0}\right)$$

where *C*_0_ is the initial concentration of adsorbed MB molecules (mg/L). The *R_L_* value is a coefficient showing the nature of adsorption: unfavorable (*R_L_* > 1), linear (*R_L_* = 1), favorable (0 < *R_L_* < 1), and irreversible (*R_L_* = 0) [66].

The Freundlich model expresses adsorption according to Equation (8):

(8) $$logq_{e}=logK_{F}+\frac{1}{n}\times logC_{e}$$

*K_F_* is the Freundlich constant related to the capacity of the adsorbent, and 1/*n* is the surface inhomogeneity parameter, a constant indicating the intensity of the sorption process.

The magnitude of 1/𝑛 qualifies the degree of heterogeneity of the adsorbent surface and indicates how favorable the adsorption process is. If 0 < 1/*n* < 1, the adsorption process is favorable, and new adsorption sites form on the surface of the adsorbent, there is a strong adsorption bond as a result of the strong intermolecular attraction. If 1/*n* > 1, it is unfavorable, and the adsorption bond between the MB molecules and the adsorbent surface is weak. If 1/*n* = 1, the process manifests as homogeneous [68]. *K_F_* and *n* can be obtained from the intercept and slope of the linear plot of ln*q_e_* versus ln*C_e_*.

The Temkin model is expressed by Equation (9):

(9) $$q_{e}=B_{T}\times lnA_{T}+B_{T}\times lnC_{e}$$

where *q_e_* is the amount of adsorbate adsorbed at equilibrium (mg/g); *C_e_* is the adsorbate concentration in the solution at equilibrium (mg/L). *B_T_* is the constant associated with the heat of adsorption and is determined by the expression *B* = R*T*/*b*, *b* is the Temkin constant (J/mol), *T* is the absolute temperature (*K*), R is the gas constant (8.314 J/mol K), and *A* is the Temkin isotherm constant (L/g). From the plot of *q_e_* versus ln*C_e_*, *B_T_* and *A* can be calculated from the slopes and intercepts, respectively. The Temkin model’s applicability indicates the adsorbate’s physical or chemical adsorption on the sorbent.

The adsorption equilibrium data were also fitted with the Dubinin-Radushkevich isotherm model according to Equation (10):

*In qe = In qD − *2*B_D_RT In (*1* + *1*/Ce)* (10)

where *qD* is the theoretical adsorption capacity at saturation, *B_D_* (mol^2^/J^2^) is the isotherm constant, *T* is the absolute temperature (K), and *R* is the universal gas constant (8.314 J/K mol) [69]. The model parameters were calculated from the *ln*(*qe*) graph versus the Polyanyi potential ε = *RT* ln (1 + 1/*Ce*). The average free energy of adsorption (*E*, kJ/mol), which characterizes the free energy of the system due to the transfer of one mole of ions from the solution to the solid surface [70], was calculated from the *B_D_* values using the following Equation (11):

(11) $$E=\frac{1}{\surd 2BD}$$

## 3. Results

### 3.1. Phase-Separated Glasses

#### 3.1.1. Structure

**Figure 1:**
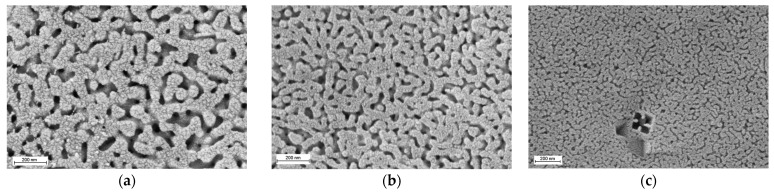
SEM images for the phase-separated glasses: (**a**) 6/55-8, (**b**) 6/60-8, (**c**) 6/65-8. Reproduced with permission from Konon et al., Glass Physics and Chemistry; published by Pleiades Publishing, Ltd., 2021 [53].

The SEM results for these iron-containing glasses have previously been published elsewhere [53]. It was found that heat treatment at 550 °C for 24 h led to the formation of a phase-separated structure with interconnected phases (Figure 1). The diameter of the liquation channels decreased with increasing SiO_2_ content.

**Figure 2:**
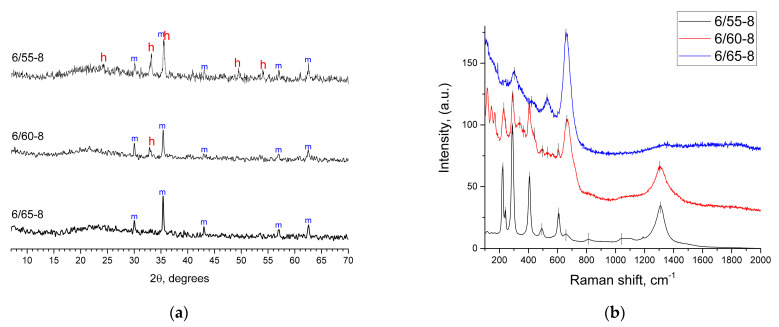
(**a**) XRPD patterns for the phase-separated glasses. The letters on the graph refer to the following phases: h—hematite (79-0006); m—magnetite (C39-1346); (**b**) Raman spectra for the phase-separated glasses.

According to the results of XRPD (Figure 2a) in glass 6/55-8, along with Fe_3_O_4_ (C 39-1346), a crystalline phase of hematite (α-Fe_2_O_3_, 79-0006) was formed. With a further increase in the silica concentration, the amount of hematite decreased. In the 6/65-8, glass magnetite was the only crystalline phase. However, the existence of crystalline hematite could be highly likely attributed to the low melting temperature of the glass batch. NBS glasses with lower SiO_2_ are known not to require high melting temperatures. However, this evidently was not the case for iron-containing glass, as 1320 °C was apparently insufficient to completely melt the raw batch of hematite (for 6/55-8 glass).

Raman spectrum of the 6/55-8 glass (Figure 2b) was a typical spectrum of hematite [71,72] containing a full set of characteristic bands at 223, 241, 289, 407, 492, 608, 661, 813, and 1310 cm^−1^. As the content of SiO_2_ increased, bands appeared at 296, 526, and 658 cm^−1^, which were attributed to magnetite [71,72]. Raman spectroscopy results are in agreement with XRPD results. The hematite and magnetite bands were much higher than the intensity of those of the glass matrix; therefore, the bands associated directly with the matrix were not observed in the recorded spectra.

#### 3.1.2. Leaching Kinetics

**Figure 3:**
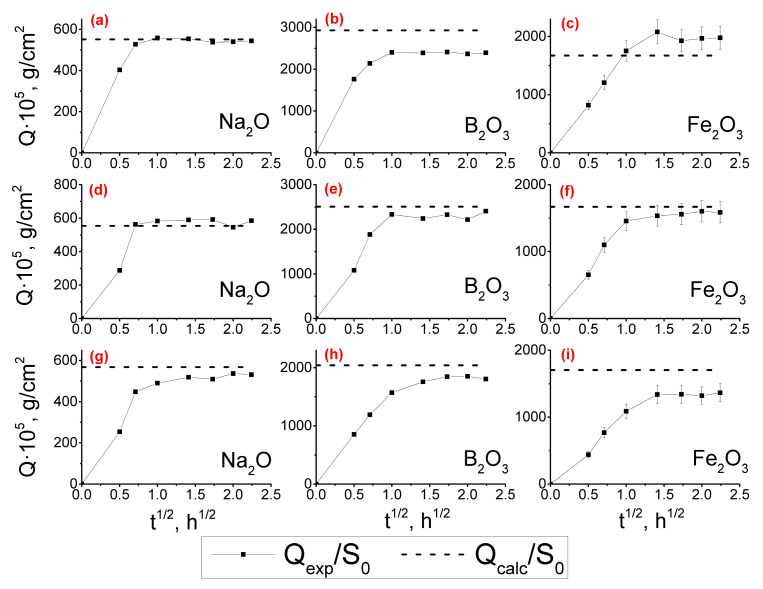
The kinetic dependencies of Na_2_O (**a**,**d**,**g**), B_2_O_3_ (**b**,**e**,**h**), and Fe_2_O_3_ (**c**,**f**,**i**) extraction into the leaching solution from phase-separated glasses 6/55-8 (**a**–**c**), 6/60-8 (**d**–**f**) and 6/65-8 (**g**–**i**).

Sodium ion exchange in NBS glasses is the fastest stage of the leaching process [73]. Therefore, the leaching rate of the phase-separated glass can be characterized through the sodium extraction kinetics. The extraction of Na_2_O from iron-containing glasses was more than 90%, and the *Q*_exp_/*S*_0_ curves reached the *Q*_calc_/*S*_0_ values (Figure 3a,d,g), indicating the completion of the leaching process and the obtaining of PGs. The kinetic dependences of the release of boron into the leaching solution are similar (Figure 3b,e,h). However, the experimental values are somewhat lower (about 80–90%) compared to the theoretically possible ones since boron ions can be partially located in the silica phase or due to their precipitation in pores. Similarly to iron-containing glasses containing 70 mol% SiO_2_ [42,73], the dependences of the component extraction rate (*Q* = *f*(√*t*)) for Na_2_O, B_2_O_3_, and Fe_2_O_3_ for the investigated glasses with lower concentrations of SiO_2_ were linear up until the values of Q_exp_/S_0_ reached a plateau close to Q_calc_/S_0_ (Figure 3). SiO_2_ practically does not get extracted from the glass into the acid-leaching solution due to the resistance of siloxane bonds in the silica-rich phase to acidic solutions and because of the polymerization and gelation processes of so-called secondary silica inside the leached glass layer [74]. Thus, silica forms the skeleton of porous glass. The percentage yield of SiO_2_ for iron-containing glasses does not exceed 10% (not shown on the graphs).

The silica content in phase-separated glass was found to not affect the effective diffusion coefficient of the Na, B, and Fe ions (the mean values are D*_Na_ ~3.3 × 10^−7^, D*_B_ и D*_Fe_ ~2.5 × 10^−7^). The introduction of Fe_2_O_3_ into the composition of the NBS glass also does not affect the D* values for all components of the glass (compared to the 6/60 glass). However, the iron-containing glass leaching rate was lower than 6/60 glass without additives.

As a result of leaching, PGs were obtained from all phase-separated glasses. As the SiO_2_ content in phase-separated glass increased, the concentration of total Fe_2_O_3_ in PGs and the proportion of FeO increased (Table 2). A slight decrease in gravimetric pore volume (from 0.45 cm^3^/g to 0.30 cm^3^/g), porosity (from 50 to 40%) (not shown in the table), and average pore diameter (from 14 nm to 5 nm) were also observed along with an increase in specific surface area (SA) (from 53 m^2^/g to 135 m^2^/g) (Table 3). The observed increase in SA and the decrease in the average pore diameter may be caused by increased silica in the boron-rich phase of the phase-separated glass. That usually leads to an increase in the number of globules of secondary silica and a decrease in the gaps between them, which form pores in PGs. The addition of Fe_2_O_3_ (compared to glass 6/60) did not affect *V*_p_, *W*, but the SA values for glasses containing Fe_2_O_3_ were 2–5 times higher than those of glass 6/60 (SA = 32 m^2^/g), and the average pore diameter was 3–8 times lower than for glass 6/60 (*d*_a_ = 41 nm).

### 3.2. Porous Glasses

#### 3.2.1. XRPD Results

**Figure 4:**
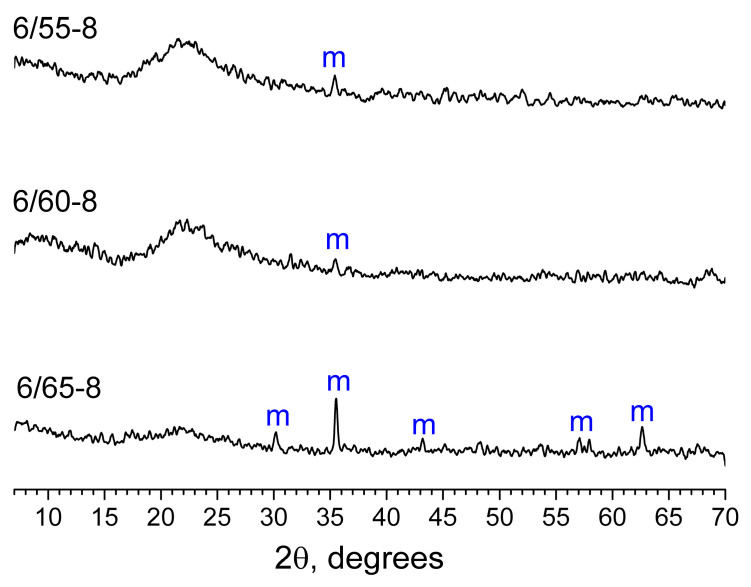
XRPD patterns for PGs obtained from phase-separated glasses 6/55-8, 6/60-8, and 6/65-8. The letter (m) on the graph refers to magnetite (C39-1346).

According to XRPD results, PGs obtained from phase-separated glasses with SiO_2_ concentrations 60 mol% and lower are predominantly X-ray amorphous; only traces of magnetite can be observed (Figure 4). The only phase-separated glass that yielded a magnetite-containing PG is 6/65-8.

#### 3.2.2. Structural Characteristics of Porous Glasses

**Figure 5:**
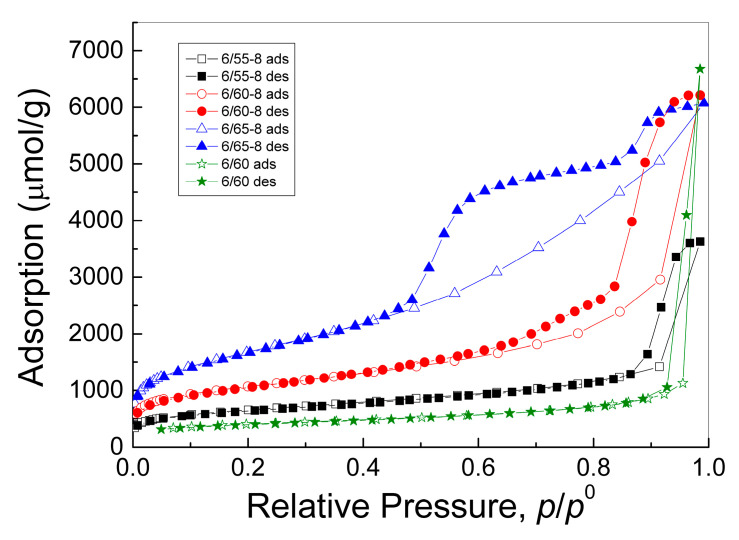
Adsorption and desorption isotherms of nitrogen at a temperature of 77 K for the investigated PGs.

Adsorption-desorption isotherms for the studied PG samples were closest to type IV according to the IUPAC classification [75] with capillary condensation characteristic of mesoporous bodies and H2-type hysteresis loop (Figure 5). The shape of the integral adsorption and desorption isotherms with a shift of the hysteresis loop towards lower relative pressures with an increase in the iron content in PG indicated that, in this case, the sizes of both the mesopores themselves and their pore necks decrease. In addition, the appearance of “steps” on the isotherms for samples with a higher silica content (6/60-8 and 6/65-8) indicated a clear polymodality of the porous structure of these samples.

**Figure 6:**
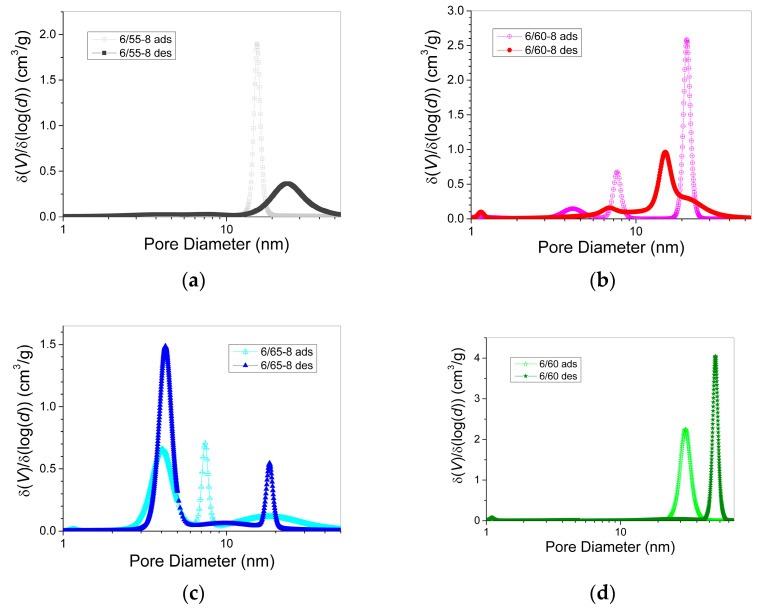
Pore diameter distribution curves for the adsorption and desorption branches of PG isotherms for glasses: 6/55-8 (**a**), 6/60-8 (**b**), 6/65-8 (**c**), 6/60 (**d**).

From the integral curves (Figure 5) using the density of liquid nitrogen (34.67 cm^3^/mol), we obtained differential curves for the pore volume distribution over their diameters (Figure 6) by approximating the adsorption and desorption isotherms using a polymodal equation of five modes. The first mode described the region of large micropores, and the remaining four modes described the region of mesopores. The regression coefficient of approximation by the polymodal equation was 0.9997–0.9999.

Figure 6 shows that all PG samples studied were characterized by a polymodal distribution of mesopores and a small number of micropores. For samples without Fe_2_O_3_ and with the least amount of silica (6-60 and 6-55-8), there was one main mode in the distribution (Figure 6a,d). As the silica content in the phase-separated glass (samples 6/60-8 and 6/65-8) increased, polymodality became evident (Figure 6b,c).

The structural characteristics of the PGs calculated from low-temperature nitrogen adsorption and desorption isotherms, including volume, specific surface area, and effective diameters of mesopores and micropores, are given in Table 3.

The total pore volume corresponds to the final value on the nitrogen adsorption isotherm at p/p^0^ = 0.985, that is, for pores with a diameter less than 133 nm. The volume of micropores determined from the first mode on the distribution curve for the adsorption isotherm is small and varies within 0.004–0.006 cm^3^/g. The mesopore volume was calculated as the difference between the total pore volume and the micropore volume.

The SA values of the studied PG samples, determined by the BET method with non-linear regression of the adsorption isotherms in the range of relative pressures p/p^0^ = 0.05–0.20, were in the range of 32–135 m^2^/g and increased with increasing silica content in the phase-separated glass (Table 3).

In general, the BET method for determining the SA of porous bodies is accurate and applicable only to mesoporous samples. The processing of samples with a microporous component by this method is not correct since this determines not only the monolayer filling of mesopores but also the volume filling of micropores. In the presence of micropores, the constant of the BET equation takes values greater than 100, indicating the presence of micropores with a high adsorption potential. For the samples studied, the constant of the BET equation took values of 200–300, which indicated the presence of micropores in PGs. The SA of only mesopores was determined by the BET method with non-linear regression of the adsorption and desorption isotherms in the range of relative pressures p/p^0^ = 0.05–0.20, taking into account the filling of micropores and with a constant in the BET Equation equal to 70, which is typical for silica adsorbents without micropores. The authors obtained this value of the BET Equation constant [62,63,64] based on the analysis of a large number of adsorption isotherms available in the literature for mesoporous silicas that do not contain micropores. The difference between the total BET-specific surface area and the mesopore-specific surface area can be considered as the micropore-specific surface area.

The effective mesopore diameters obtained by approximating the equilibrium adsorption and desorption isotherms with a polymodal dependence are given in Table 3. Volume fractions of each mode in the total volume of mesopores are given in parentheses. Considering each mode’s contribution to the mesopore distribution curve, the average mesopore diameter for equilibrium adsorption and desorption was calculated.

Knowing the average diameter *d_a_*, the specific surface area (*S*), and the mesopore volume *V_mes_*, one can calculate the structural coefficient *K* using Equation (12):

*K = d_a_ S/V_mes_* (12)

which characterizes the predominant pore shape. For plane-parallel pores, this coefficient is 2, for cylindrical pores-4, and for spherical pores-6. For interglobular pores formed by regular packings of secondary silica globules with coordination numbers (i.e., the number of contacts of globules) from 4 to 12, the structural coefficient is *K* = 2.8 ± 0.2 [64]. The structural coefficient *K* for the studied iron-containing glasses samples decreased from 4.8 to 3.1 with an increase in the silica content in the phase-separated glass, indicating a change in the shape of the pores from cylindrical with spherical elements to interglobular. For micropores with an average diameter of 1.1 nm, the structural coefficient *K* was in the range of 2.4–2.9, which corresponds mainly to interglobular pores.

Analysis of the data given in Table 3 showed that with an increase in the iron concentration in PG and silica content in phase-separated glass from sample 6/55-8 to sample 6/65-8, the SA of mesopores increases from 39–123 m^2^/g and, accordingly, the total BET surface increases from 53–135 m^2^/g, the average size of the mesopores decreases from 14 to 5 nm. The fact that, upon switching from the adsorption branch to the desorption branch, which mainly characterizes the size of the pore necks, the average size of mesopores either slightly increased or decreased (Table 3) indicated the contribution of cone-shaped and bottle-shaped pores. This was also evidenced by the shift of the maxima of the main peaks on the curve of the differential distribution of mesopores (Figure 6) for the adsorption and desorption branches either towards a decrease or towards an increase in pore sizes.

When comparing glasses 6/60 and 6/60-8 with the same amount of SiO_2_ in the phase-separated glass before leaching, it can be seen that the introduction of iron leads to a decrease in total pore volume, an increase in the specific surface of the pores, and a sharp decrease in average mesopore size with the appearance of a pronounced polymodality. Fe_2_O_3_ extraction into the acid solution during leaching reached 96%, indicating that the majority of all iron ions were present in the chemically unstable boron-rich phase. The distribution of the Na_2_O and B_2_O_3_ in this phase and their subsequent leaching form the shape and spread of the secondary silica inside the pore space of the porous glass. Hence, it can be assumed that the introduction of Fe_2_O_3_ assisted in the formation of secondary silica globules of smaller sizes and different diameters, which led to a decrease in the average size of mesopores and the appearance of polymodality.

#### 3.2.3. Sorption of Methylene Blue

**Figure 7:**
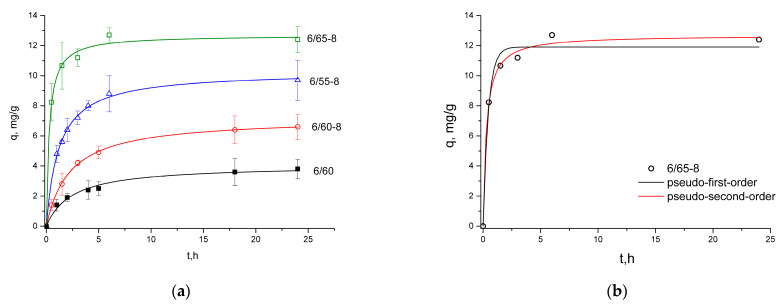
Adsorption kinetics of MB for all of the samples fitted with PSO model (**a**), and kinetic curves for the sample 6/65-8 fitted with both PFO and PSO models (**b**).

The kinetic data were analyzed using the PFO and PSO kinetic Equations (3) and (4), respectively, and their corresponding parameters were summarized in Table 4. The values of correlation coefficients (*R*^2^) obtained from pseudo-second-order kinetics were higher (>0.99) than those from pseudo-first-order kinetics. Thus, pseudo-second-order kinetics was a better fit to describe the process of MB adsorption on PG samples (Figure 7).

An 80% saturation of the sorbent by MB occurred approximately after 6 h (Figure 6).

**Figure 8:**
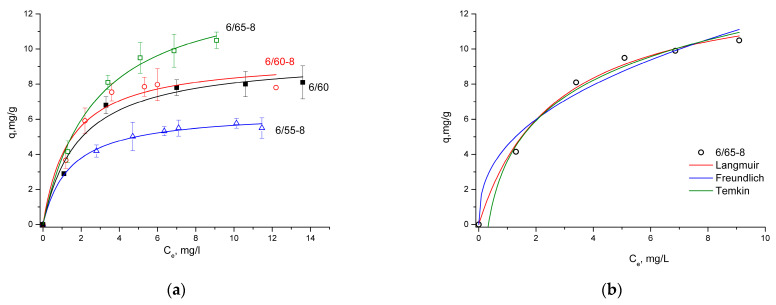
Equilibrium isotherms fitted with the Langmuir model for all investigated samples (**a**); equilibrium isotherms fitted with the Langmuir, Freundlich, and Temkin models for glass 6/65-8 (**b**).

Adsorption isotherms were fitted by Langmuir, Freundlich, Temkin, and Dubinin-Radushkevich models (Figure 8). As can be seen from the data summarized in Table 5, the R^2^ values calculated from the Langmuir isotherm model were higher than those calculated from the Freundlich isotherm equation, indicating that the Langmuir model was more suitable to interpret MB adsorption on the synthesized porous glasses, suggesting monolayer adsorption on homogeneous surfaces with identical sites [69]. The *R*_L_ values were between 0.07 and 0.12, which falls in the range of 0 < *R*_L_ < 1, which implied that the adsorption process was favorable and that the system could be considered appropriate for the adsorption of MB.

## 4. Discussion

Studying adsorption kinetics can provide information on the adsorption mechanism [76]. The kinetic mechanism of the adsorption process consists of three consecutive steps of mass transport. The first step is film diffusion (external diffusion), which is the transfer of adsorbate molecules from the bulk of the solution to the outer surface of the adsorbent. The second one is intra-particle diffusion, where the adsorbate molecules transfer from the outer surface of the adsorbent into the pores. In the final step, the dye is adsorbed into the active sites at the interior of the adsorbent particle. Various kinetic models are used to assess the adsorption data, such as the pseudo-first-order (PFO) Equation (3), the pseudo-second-order (PSO) Equation (4), and the intra-particle diffusion Equation (5) [66]. The PFO and PSO models describe the entire process, from diffusion in the external film to intra-particle diffusion and interaction between the adsorbate and functional groups of the adsorbent. The intra-particle diffusion is described by a separate equation [69]. A pseudo-first-order model describes dye adsorption occurring through mass transfer or physisorption (physical adsorption) process, while pseudo-second-order kinetics suggest chemical interactions occur [77].

As was evident from the correlation coefficients (*R*^2^) and the calculated sorption capacity values (Table 4), the sorption of MB for the investigated glasses fits better with the pseudo-second-order model (Figure 7a,b), which infers chemisorption [20]. Methylene blue is a cationic dye; hence, it is positively charged, which means that it would readily attach to the porous glass’s negatively charged Si-O− surface. Oxygen-containing groups, namely hydroxyl groups (–OH) connected with the Si surface of the inner pore space of PGs, provide affinity (binding) sites for MB adsorption [20,78]. The pseudo-second-order kinetic model assumes chemical adsorption as the rate-determining step [77,78,79].

According to the intra-particle diffusion model, the sorption capacity (*q*_t_) dependence on the square root of time (t^0.5^) should be linear. If intra-particle diffusion is involved in the adsorption process and the graph passes through the origin, then intra-particle diffusion is the limiting stage of the process. This means that diffusion inside the particles is the only mechanism that controls the sorption process. On the other hand, if multiline plots are obtained, this indicates that intra-particle diffusion is not the only rate-regulating step. The *k*_id_ values for intra-particle diffusion are obtained from the slope of the straight sections of the plot of *q*_t_ versus t^0.5^. If the graph has two straight sections, diffusion is characterized by two rate constants, *k*_id1_ and *k*_id2_. If the values of *k*_id1_ are higher than the values of *k*_id2_, then one speaks of the limitation of available free places for the diffusion of molecules and blockage of pores [67,80].

**Figure 9:**
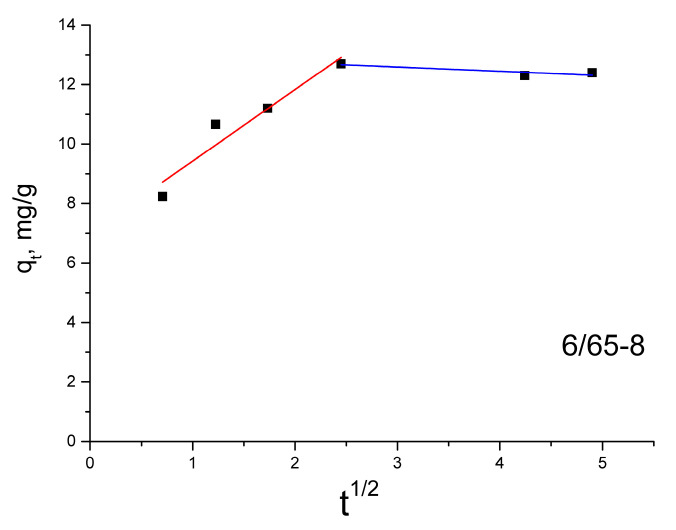
Intra-particle diffusion model for the glass sample 6/65-8.

The dependence of the sorption capacity on the square root of time for the 6/65-8 sample (Figure 9) illustrates the fitting of the intra-particle diffusion model typical for all the investigated glass samples. All of the graphs are non-linear, have two plots, and do not pass through the origin. This indicates that intra-particle diffusion is not the only rate-controlling step, and MB adsorption on samples is a complex process. Other factors, such as surface area, pore size, and volume, can affect the adsorption rate [81]. The values of *k*_id1_ are higher than the values of *k*_id2_ (Table 4), which indicates that the limitation of available free places for the diffusion of molecules and blockage of pores takes place [66,67,80]. This is consistent with the existence of cone-shaped and bottle-shaped pores established from the nitrogen adsorption and desorption isotherms. The “bottlenecks” in the bottle-shaped pores are narrower than the rest of the pore body, which might cause the blockage, prevailing the dye from transferring deeper inside the pore space.

The adsorption isotherm is important in understanding the interactions between solutes and adsorbents, as it indicates how adsorbed molecules distribute themselves between the liquid and solid phases until the adsorption process reaches an equilibrium state [76]. Different isotherm models are used to analyze the adsorption equilibrium data. They are a valuable tool for reliable prediction of the adsorption process and quantitative comparison of adsorption behaviors [69,76]. The models used to analyze the data in this work differ from each other in how the heat of adsorption varies with surface coverage: the Langmuir isotherm does not assume that the heat of adsorption decreases as surface coverage increases; the Freundlich suggests a logarithmic decrease, while Temkin suggests a linear decrease.

For the investigated PGs, the Langmuir model was more suitable for interpreting the adsorption of MB (Table 5, Figure 8) than the Freundlich isotherm. The Langmuir model was applied to estimate the maximum sorption capacity corresponding to a monolayer adsorbent coating. The monolayer adsorption process means that no further adsorption occurs once adsorption takes place at specific sites on the adsorbent. Therefore, the adsorption is closely related to the surface area of the adsorbent and the driving force, such as the London-van der Waals force [76]. The maximum sorption capacity was demonstrated by the glass sample 6/65-8 (10.5 mg/g). This glass shows the highest SA value, previously attributed to increased adsorption capacities [77,82].

The Freundlich isotherm is a classic model for multilayer adsorption on heterogeneous adsorbent surfaces and adsorption sites with different energies [66,69]. The Temkin isotherm equation also implies a heterogeneous surface. It assumes an adsorbent–adsorbate interaction that causes a linear decrease in the heat of adsorption of all molecules in the coated layer. That adsorption is characterized by a uniform distribution of binding energies up to a certain maximum binding energy [69,83]. The correlation coefficient for the Temkin model is also high for all samples (0.93–0.99); the low values of the calculated coefficient *b* in the Temkin model indicate physical adsorption. The *R*^2^ values of the Temkin model indicate that it potentially could have been applied to describe the adsorption process; however, the suitability of the Langmuir model indicates a homogeneous surface of the sorbent. The values of the *b* coefficient also do not agree with the better-fitting PSO model, which infers chemisorption.

**Figure 10:**
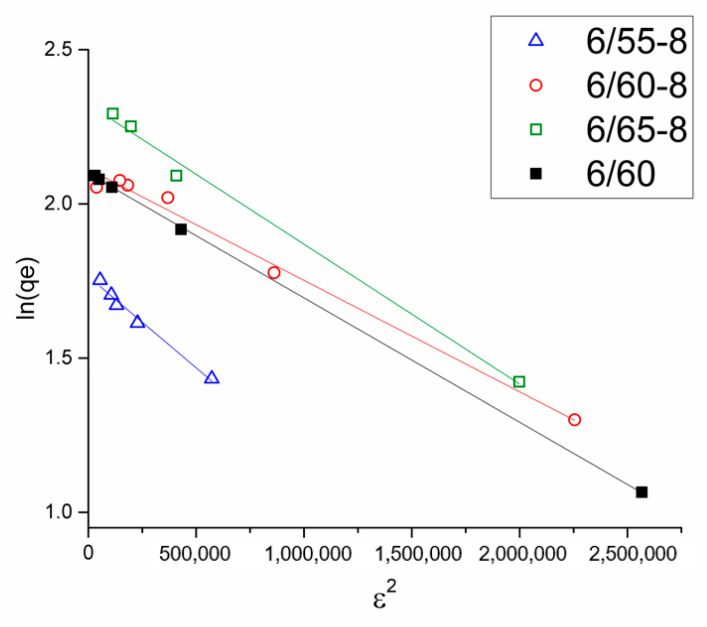
Dubinin-Radushkevich model for all the glass samples.

The Dubinin-Radushkevich model is often used to distinguish physical and chemical adsorptions (Figure 10). The correlation coefficient value for this model (Table 5) indicates that the experimental data are consistent with it. The *E* value is a general indicator of the type of adsorption and is a useful tool for gaining insight into the nature of the interactions between the adsorbate and active centers on the surface of the adsorbent [70]. *E* values less than 8 kJ/mol indicate physical adsorption. When the *E* values are between 8 and 16 kJ/mol, the process proceeds according to ion exchange theory. Chemisorption is observed at *E* values in the range of 20–40 kJ/mol [70]. The *E* values obtained in this study are less than 8 kJ/mol, suggesting that the adsorption of MB in the samples is physical adsorption. These findings contradict the PSO model, which was a better fit for the kinetic data. However, the high values of the R^2^ coefficients for both the PSO model and the Dubinin-Radushkevich model led us to believe that MB adsorption on the investigated samples is a complex process that consists of multiple stages, including both physical and chemical adsorption.

Overall, the MB sorption capacity values of the investigated PGs are of the same order of magnitude as some other porous glasses and materials (Table 6).

## 5. Conclusions

Three compositions of phase-separated Fe_2_O_3_-containing NBS glasses with different SiO_2_ concentrations were investigated by XRPD and Raman spectroscopy. The leaching kinetics of these glasses in an aqueous 3M HCl solution at boiling was studied. As a result of the through-leaching of the phase-separated glasses, porous glasses were obtained. The porous structural characteristics of those PGs were studied by the classical gas adsorption method, using the equilibrium adsorption and desorption isotherms of nitrogen at the liquid nitrogen temperature. The sorption properties of the obtained PGs were studied with respect to methylene blue.

The only porous glass that contains magnetite after leaching is the one with the highest concentration of SiO_2_ in phase-separated glass (before acid treatment). Analysis of the nitrogen adsorption-desorption isotherms demonstrated that all PGs obtained were characterized by a polymodal distribution of mesopores and a small number of micropores. With increasing silica content in the phase-separated glass, an increase in the specific surface area and a decrease in the average pore diameter were observed, along with the changes in the shape of the mesopores from cylindrical with spherical elements to interglobular. Micropores were found to be mainly interglobular.

The sorption of MB for the investigated glasses complies with the pseudo-second-order model, which suggests the chemisorption of the positively charged methylene blue onto a negatively charged Si-O^−^ surface of the porous glass. T The equilibrium isotherms for the investigated PGs follows the Langmuir model, which suggests that adsorption occurs in a monolayer pattern on homogeneous adsorbent sites. The equilibrium isotherms were also well-fitted with the Dubinin-Radushkevich model. A sorption energy value lower than 8 kJ/mol suggested physical adsorption. The MB adsorption on the investigated samples was established to be a complex process consisting of multiple stages, including physical and chemical adsorption.

The maximum sorption capacity (10.5 mg/g) was demonstrated by glass sample 6/65-8 with the highest specific surface area value. The MB sorption capacity values of the investigated PGs are of the same order of magnitude as those of some other porous glasses and porous materials.

## 6. Patents

An application for a patent for an invention was sent to the Federal Institute of Industrial Property (Russia) on 27 June 2022. The decision to grant patent RU 2022117542 titled “Method for producing magnetite-containing porous glass” (authors: Konon M., Anfimova I., Polyakova I., Antropova T., applicant Grebenshchikov Institute of Silicate Chemistry, Russian Academy of Sciences) was made on 1 February 2023.

## Figures and Tables

**Table 1:** Composition, density, and volumetric concentration of the glasses. Compositions and density values are adapted from [53].

Glass Designation *	Glass Composition As-Analyzed, wt% **				Volumetric Concentration *C*_v_, g/cm^3^				Density ρ, g/cm^3^
	SiO_2_	B_2_O_3_	Na_2_O	Fe_2_O_3_ ***	SiO_2_	B_2_O_3_	Na_2_O	Fe_2_O_3_	
6/60	57.39	36.98	5.63	-	1.23	0.79	0.12	-	2.145
6/55-8	47.65	29.75	5.61	16.99	1.15	0.72	0.13	0.41	2.415
6/60-8	51.93	25.46	5.64	16.97	1.26	0.62	0.14	0.41	2.437
6/65-8	56.58	20.55	5.72	17.14	1.40	0.51	0.14	0.42	2.470

* The numbers in the designation correspond first—to sodium oxide content, second through the fraction to silicon oxide, and last through the hyphen to the content of Fe_2_O_3_ according to the synthesis, in mol%. ** Unlike glass designation, glass compositionsAs-Analyzed in wt% because wt% is required for *C*_v_ calculation. *** in terms of Fe_2_O_3_.

**Table 2:** Effective diffusion coefficient (D*, cm^2^/s), glass components extraction into the leaching solution (%), and composition of PG as-analyzed (wt%).

Glass	D*, cm^2^/s *			Component Extraction, %				PGs Composition (as Analyzed, wt%)				
	Na	B	Fe	Na_2_O	B_2_O_3_	SiO_2_	Fe_2_O_3_	SiO_2_	B_2_O_3_	Na_2_O	Fe_2_O_3_	FeO
6/60	3.8 × 10^−7^	2.1 × 10^−7^	-	100	81	11	-	87.05	12.73	0.22	-	-
6/55-8	3.6 × 10^−7^	2.4 × 10^−7^	3.5 × 10^−7^	100	82	8	100	85.88	11.25	0.59	1.97	0.30
6/60-8	3.7 × 10^−7^	3.1 × 10^−7^	2.7 × 10^−7^	100	96	10	96	87.20	6.95	0.35	5.16	0.34
6/65-8	2.7 × 10^−7^	2.1 × 10^−7^	1.4 × 10^−7^	94	91	5	80	82.97	5.38	0.40	9.75	1.49

* D* values were determined for the time interval of 1 h after the start of the experiment.

**Table 3:** Structural characteristics of the studied PGs.

PG Sample	6/55-8	6/60-8	6/65-8	6/60
Total pore volume, cm^3^/g	0.126	0.215	0.211	0.231
Micropore volume, cm^3^/g (percentage of the total volume is given in parentheses)	0.0058 (4.6%)	0.0045 (2.1%)	0.0058 (2.8%)	0.0039 (1.7%)
Mesopore volume, cm^3^/g	0.120	0.210	0.205	0.227
BET-specific surface area, m^2^/g	53	86	135	32
BET-specific surface area of mesopores, m^2^/g	39	63	123	22
Specific surface area of micropores, m^2^/g	14	23	12	10
Effective mesopore diameters, nm (adsorption isotherm, the volume fraction of the mode in mesopore volume is given in parentheses)	3.22 (0.7%) 15.4 (78.9%) 16.3 (2.5%) 17.1 (17.9%)	4.16 (11.7%) 7.68 (20.2%) 20.3 (57.8%) 21.2 (10.3%)	4.04 (53.1%) 7.33 (15.2%) 16.8 (31.3%) 24.5 (0.4%)	26.3 (74.9%) 54.6 (25.1%)
Effective mesopore diameters, nm (desorption isotherm, the volume fraction of the mode in mesopore volume is given in parentheses)	4.00 (11.9%) 8.03 (4.9%) 23.3 (69.8%) 33.3 (13.4%)	6.87 (4.3%) 10.9 (34.0%) 15.0 (34.1%) 20.3 (27.6%)	2.86 (1.7%) 4.19 (62.1%) 5.51 (16.1%) 17.1 (20.1%)	5.12 (1.9%) 25.2 (6.9%) 50.8 (91.2%)
Average mesopore diameter, nm (adsorption)	15.3	11.4	5.84	30.2
Average mesopore diameter, nm (desorption)	14.3	13.6	5.12	41.1
Average micropore diameter, nm (adsorption)	1.07	1.11	1.15	1.10
Average structural coefficient of mesopores	4.79	3.92	3.13	4.25
Average structural coefficient of micropores	2.59	2.43	2.88	2.43

**Table 4:** The comparison of kinetic parameters of MB for the investigated porous glass samples.

Glass		6/55-8	6/60-8	6/65-8	6/60
	q_exp_	9.70 ± 1.34	6.60 ± 0.85	12.40 ± 0.86	3.80 ± 0.65
Pseudo-first-order	q_calc_	9.16	6.42	11.91	3.62
	k_1_, min^−1^	0.61	0.35	2.20	0.31
	R^2^	0.9804	0.9882	0.9772	0.9498
Pseudo-second-order	q_calc_	10.29	7.20	12.70	4.06
	k_2_, g/mg min	0.08	0.06	0.28	0.10
	R^2^	0.9978	0.9991	0.9942	0.9842
Intraparticle diffusion	k_id1_	2.77	2.73	2.41	0.89
	C	2.26	0.54	7.02	0.56
	R^2^_1_	0.9749	0.9999	0.8888	0.9712
	k_id2_	0.35	0.66	0.14	0.50
	C	7.92	3.45	13.02	1.39
	R^2^_2_	0.9228	0.9659	0.5329	0.9818

**Table 5:** Parameters calculated from isotherm equations for MB on PGs.

Glass		6/55-8	6/60-8	6/65-8	6/60
q_exp_, mg/g		5.50 ± 0.58	7.80 ± 0.91	10.50 ± 0.47	8.10 ± 0.94
Langmuir Equation	q_m,_ mg/g	6.44	9.52	13.90	9.58
	K_L_, L/mg	0.71	0.72	0.38	0.53
	R^2^	0.9952	0.9620	0.9930	0.9765
	R_L_	0.07	0.08	0.12	0.08
Freundlich Equation	1/n	0.19	0.24	0.41	0.30
	K_F,_ L/g	3.64	4.80	4.51	3.95
	R^2^	0.9886	0.8990	0.9691	0.9239
Temkin Equation	B_T_	1.01	1.85	3.32	2.04
	A, L/g	26.93	10.01	2.97	5.20
	b, kJ/mol	2.45	1.34	0,75	1,21
	R^2^	0.9906	0.9264	0.9893	0.9554
Dubinin-Radushkevich Equation	q_D_, mg/g	5.777	8.275	10.203	8.153
	B_D_, mol^2^/J^2^	1.187 · 10^−4^	1.514 · 10^−4^	0.447 · 10^−4^	0.187 · 10^−4^
	R^2^	0.9791	0.9874	0.9910	0.9998
	E, kJ/mol	0.064	0.057	0.105	0.163

**Table 6:** Maximum adsorption capacity of MB for various adsorbents.

Adsorbent	Adsorption, mg/g	Ref.
Porous glasses	4.90–20.69	[76,78]
Raw zeolite	6.10	[84]
Raw kaolin	13.99	[85]
Magnetic multi-wall carbon nanotube	15.87	[86]
Magnetite-containing porous glass	10.5	This study

## Data Availability

Not applicable.
